# Ongoing Commercialization of Gestational Surrogacy due to Globalization of the Reproductive Market before and after the Pandemic

**DOI:** 10.1007/s41649-022-00215-4

**Published:** 2022-08-18

**Authors:** Yuri Hibino

**Affiliations:** College of Philosophy in Interdisciplinary Sciences, Kanazawa University, Kanazawa City, Japan

**Keywords:** Reproductive ethics, Gestational surrogacy, Commercialization, India, Thailand

## Abstract

Surrogacy tourism in Asian countries has surged in recent decades due to affordable prices and favourable regulations. Although it has recently been banned in many countries, it is still carried out illegally across borders. With demand for surrogacy in developed countries increasing and economically vulnerable Asian women lured by lucrative compensation, there are efforts by guest countries to ease the strict surrogacy regulations in host countries. Despite a shift toward “altruistic surrogacy”, commercial surrogacy persists. Recent research carried out by international organizations that seek to establish a legal relationship between the commissioning parents and children in cross-border surrogacy arrangements, under the guise of the “best interests of the child,” appears to promote a resurgence of overseas commercial surrogacy rather than restrict it. Further commercialization of surrogacy should be prevented by carefully investigating the reality of the surrogacy process.

## Introduction

Gestational surrogacy is an attractive option for those who wish to have children. If you can access IVF surrogacy without limitation (in combination with access to healthy sperm and eggs), you can definitely have a baby. Consequently, reproductive technologies can satisfy a desire that adoption cannot (Choudhury 2016). In developed countries, surrogacy is increasingly used by wealthy people with liberal ideas to achieve a desired lifestyle and build intimate relationships. “Procreative consciousness” among LGBT individuals has accelerated global demand for surrogacy (Berkowitz 2007). The use of surrogacy by individuals from developed countries has been forced onto emerging Asian countries such as India, Thailand, and Cambodia and Mexico (Nahavandi 2016; Shetty 2012), making the exploitation of vulnerable groups a concern (Kirby 2014; Crozier 2014; Orfali and Chiappory 2014). Surrogacy tourism in many of these countries has been shut down in recent years following scandals that triggered the exclusion of foreign nationals.

Surrogacy practices have become more commercialized in recent years despite being called “altruistic”. Networks of global surrogacy markets have emerged, and established surrogacy stakeholders are influencing research and the public policy decision-making processes. International organizations such as the United Nations (UN) and the Human Rights Consultative Committee (HRCC) have begun to address cross-border surrogacy through research projects. Border blockages during the 2020 COVID-19 pandemic brought a more intense focus to this contested issue (Fronek and Rotabi 2020).

In this paper, I posit that the commercialization of surrogacy has grown out of the surrogacy tourism boom in Asia and that its influence has impacted the policies of developed countries. I conclude that de-facto commercialization should be stopped by carefully investigating the reality of the surrogacy process.

## Types of Surrogacy

According to a survey conducted by Cornell Law School, the International Human Rights Policy Advocacy Clinic and the National Law University, surrogacy is completely banned in 50 countries and is legal in 40 countries. There are no regulations regarding surrogacy in 30 countries, while for 72 countries, there is no information made available (Kalantry et al. 2017). In 21 countries, surrogacy is partially tolerated.

Surrogacy is generally dichotomized into “commercial” and “altruistic” (or non-commercial) surrogacy. Altruistic surrogacy is based on a “gift relationship” motivated by love or altruism in which a woman—often a close friend or relative—agrees to have a child for an infertile couple. On the other hand, commercial surrogacy is modelled on a business relationship. Both parties are (or are expected to be) motivated by personal gain to enter into a legally enforceable agreement, which stipulates that the contract mother is to bear a child for the intending (or “commissioning”) parents in exchange for a fee (van Zyl and Walker 2013).

Altruistic surrogacy is found in the Surrogacy Arrangement Act 1985 in the UK, which was the first country in the world to regulate surrogacy. Surrogate mothers are not permitted to receive compensation other than for necessary expenses. Altruistic surrogacy is typically found in countries that do not allow commercial surrogacy.

Commercial surrogacy is allowed in several states in the USA. The federal government has a liberal attitude toward the reproductive industry and does not legally regulate it. As a result, surrogacy is viewed as a legal business transaction in several states, including California.

In general, there are two ways to establish a legal relationship between a commissioning parent and a child born through surrogacy. The first way involves the surrogate mother being the legal mother in the first instance, with the commissioning parent becoming the legal parent after a “parental order” court decision. This method was first adopted in the UK. Under this framework, the surrogate mother can change her mind after giving birth and choose not to hand the child over to the commissioning parent(s) (Olaye-Felix et al. 2022). The second way is for the individual(s) who requested surrogacy to be legal parent(s). For example, in India, the commissioning parent’s names are listed on the child’s birth certificate in the first instance (Indian Council of Medical Research 2005). Surrogate mothers have no rights to the children and the commissioning parent(s) immediately become the legal parent(s). Since no court decision was required, foreign commissioning parents were able to quickly obtain the necessary documents for the child. Countries that adopt a commercial model often use this method.

## Methods

**Fig. 1:**
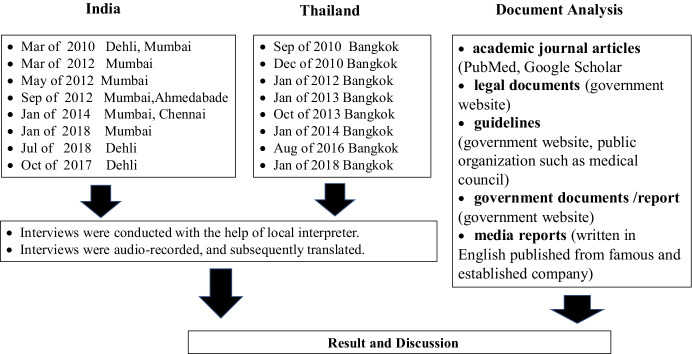
Research Methods

This paper is based on published documents and fieldwork (Fig. 1). The documents include academic journal articles, legal documents, guidelines, government documents, and media reports. Fieldwork was conducted on several occasions between 2010 and 2018 in Asian countries including India, Thailand, Vietnam, and Cambodia. This took the form of interviews with local doctors, brokers, policymakers, surrogate mothers, egg donors, and prospective parents. In this study, field data from India and Thailand has been analysed and cited. Observation and anecdotal evidence from other local sites has also been incorporated into the analysis and discussion.

Fieldwork in India was conducted from 2010 to 2018. During this period, the author visited India eight times and stayed in several regions, including Mumbai, Delhi, Chennai, Anand, and Ahmedabad. The most recent fieldwork was conducted in Mumbai in January 2018 and in Delhi in July and October of 2018 and focused on people’s views regarding altruistic surrogacy and the Surrogacy (Regulation) Bill. Views regarding altruistic surrogacy (as proposed in the 2016 Surrogacy (Regulation) Bill) and current surrogacy practices by local practitioners were also explored. At the time of last visit, the author’s informants included two policymakers, three non-government organization (NGO) members, one journalist, two doctors, and one surrogate broker who was formerly a surrogate herself.

Fieldwork in Thailand was conducted from 2010 to 2018. During this period, the author visited Thailand eight times and stayed in Bangkok on each occasion. The most recent visit was in January 2018. The fieldwork primarily focused on views regarding non-commercial surrogacy and the new Protection of Children Born Through Assisted Reproductive Technologies Act. Current surrogacy practices both in Thailand and neighbouring countries were also explored. At the time of last visit, the author’s informants included one lawyer, one researcher, two doctors, two policymakers, and one surrogate broker who was formerly a surrogate herself.

On-site, face-to-face interviews were supplemented with interviews conducted over Skype when necessary. Interviews were conducted in English or in the local language with assistance from interpreters, as applicable. Most interviews were audio-recorded, and interviews conducted in local languages were transcribed and subsequently translated for confirmation. Transcripts are cited in this paper to illustrate the findings. Citations of interview data are presented without identifying information to protect the privacy of participants. Approval for this study was obtained from the Institutional Review Board, numbers 1136 and 1048 refer.

## Surrogacy Tourism in Asia

In vitro fertilization (IVF) has been performed in Asian countries for several decades since it was first successfully performed in the UK in 1978 (Johnson 2019). In some Asian countries, third party reproduction derived from IVF—such as egg donation, surrogacy, and sex selection—has been offered under medical tourism promotion policies. They became particularly big industries in India and Thailand (Pande 2011; Whittaker 2016b).

In India, commercial surrogacy has been permitted since 2002. The country’s affordable surrogacy programmes have attracted clients from all over the world. A number of scandals emerged while the bill to legalize commercial surrogacy was under development, resulting in a ban on foreign surrogacy in 2015 (Mahapatra 2008; Desai 2012).

The low cost of surrogacy in India stimulated massive global demand. Thus, when India began to limit surrogacy tourism in 2012, new markets developed. Thailand emerged as the most attractive alternative destination to India. Scandals soon followed in Thailand in the summer of 2014, and a new Thai law to regulate assisted reproductive technologies was later enacted in January 2015 (Murdoch 2014; Lloyd Parry 2014).

The impact of the ban in Thailand spreads to neighbouring countries. In September 2014, an IVF laboratory was established at a clinic in Phnom Penh, Cambodia, and commercial surrogacy was offered in collaboration with a Thai clinic. The Cambodian government, however, declared in November 2015 that it regarded commercial surrogacy as human trafficking, and in October 2016, surrogacy was banned by a decree of the Ministry of Health (Article 12 of “Blood, Ovum, Born Marrow and Cell Control Ordinance”). While crackdowns continued thereafter (BBC 2017a, b; Handley 2018), it has been reported that meetings have been held between ministries to legalize altruistic surrogacy for Cambodian locals (DPA 2017; Vida 2018).

After the ban in Cambodia, Thai clinics expanded to Laos and Myanmar, where government crackdowns followed (BBC 2017a, b; Head 2018). Although surrogacy tourism has been banned in many Asian countries, it is still conducted illegally across borders where economically vulnerable Asian women are used as surrogate mothers (Condon 2016; Attawet 2022).

In short, since the shutdown of the surrogacy market in India, the demand for surrogacy services has been met by other markets in countries such as Thailand, Mexico, Nepal, and Cambodia. The surge in concentration of foreign customers over such a short period of time resulted in scandals related to exploitation, human trafficking, and child abandonment (Roth 2020; Meyers-Belkin 2020). Consequently, Thailand implemented its ban in 2014, followed by Nepal in 2015, Mexico in 2016, and Cambodia in 2016.

Market demands then shifted to Eastern Europe, where surrogacy is legal in Russia, the Ukraine, and Georgia and is not prohibited in some other Commonwealth of Independent States (CIS) countries. Due to the border blockade resultant of the 2020 COVID-19 pandemic, it was reported that foreign clients could not enter the region and that babies were left behind. This triggered more active local anti-surrogacy movements and calls for measures to exclude foreign nationals or prohibit surrogacy altogether (Ukrinform 2020). Despite this, new markets for commercial surrogacy are potentially emerging elsewhere in places where there is no restrictive regulation.

## The Reality of Altruistic Surrogacy in Asia: the Cases of India and Thailand

### India

In India, foreign nationals have been banned from engaging in commercial surrogacy since 2015. The Surrogacy [Regulation] Bill was proposed in 2016 to ban commercial surrogacy for foreign clients while allowing altruistic surrogacy between relatives for Indian couples. Under the proposed bill, the commissioning couple must be Indian and have been legally married for more than 5 years. The surrogate mother must be a close relative of the infertile couple and can only become a surrogate mother on one occasion (Timms 2018a, b).

The bill was criticized by stakeholders for being too restrictive (Photopoulos 2017). For example, Dr. Nayna Patel, head of the Akanksha IVF clinic in Anand, Gujarat, who is known for running a large surrogacy house, opposed the bill and gathered surrogate mothers to protest against it (Bedi 2016). It was argued that the commercial surrogacy ban would deprive working-class women and their families of substantial income streams (Arvidsoon et al. 2017; Pande 2013). Dr. Patel claimed that only 25 out of every 1000 surrogacy cases she handled involved close relatives as surrogate mothers.

Feminists have also spoken out against the bill, saying that altruistic surrogacy between relatives reflects a patriarchal kinship model (Amar 2017). Sama Resource Group for Women and Health, an advocacy organization for women’s health, describes finding a surrogate mother in a relative as old-fashioned nostalgia. In reality, this practice raises the potential for the coercion of vulnerable women within the family structure. According to Sama, excessive surrogacy restrictions will inevitably lead to black markets in Indian society (Nadimpally et al. 2016; Rudrappa 2017; BMJ 2019; Trompenaars and Hampden-Turner 1997).

An ex-surrogate who worked for a surrogate and egg donor broker in Mumbai explained her stance against altruistic surrogacy, as follows (2016, personal communication):

Altruistic surrogacy between relatives is difficult in India. Infertile couples are reluctant to ask their relatives for a surrogate mother because infertility is embarrassing, and they hate to be known to their privacy. They prefer to ask a third party who is unknown (that is, they are willing to paying for the surrogate). For poor women, their normal work can't make that much money. Foreign clients are more welcomed because they can pay more than Indians and she can get extra money. In my opinion, whether commercial or altruistic is indifferent. It's almost the same thing, isn’t it? And even if the government bans it, we can do most of the things with bribes in this society.

One ex-surrogate broker spoke of plans to send her surrogate mothers abroad to escape domestic regulations. The hypothesis that the tightening of regulations will lead to the emergence of black markets is therefore becoming a reality (Rudrappa 2016). Once the commercial surrogacy market emerged, the option of regulation and proper implementation seemed appropriate.

After significant revisions, the 2016 bill was finalized in 2019 and approved by the parliament in February 2020. While the altruistic surrogacy principle was retained within the revised Surrogacy (Regulation) Bill 2020, the requirement that the surrogate mother be a close relative of the infertile couple was removed. Now, any “willing woman” could become a surrogate mother. With the provision of relatedness removed, working-class women are overwhelmingly disadvantaged (Rozée et al. 2020) and will inevitably become surrogate mothers. Banning commercial surrogacy for foreign nationals simply shifted the problem of exploiting surrogate mothers to local populations.

Finally, the Surrogacy (Regulation) Act 2021 came into effect from January 2022. The Act aims to prohibit commercial surrogacy but allows for altruistic surrogacy. In stark contrast to the bill that was approved by parliament in 2020, which defined that “any willing women” can be a surrogate mother, the Act 2021 reverts to the earlier proposal that altruistic surrogacy should be conducted between close relatives and has stipulated it as such. Consequently, access to surrogacy services for infertile Indian heterosexual couples is limited. We do not yet know how altruistic surrogacy under the Act 2021 is being conducted in reality in India, and this should be explored.

### Thailand

A 2002 guideline issued by the Medical Council of Thailand that banned commercial gamete donation and surrogacy was not binding (Medical Council of Thailand 2002; Virutamasen et al. 2001; Whittaker 2016a). Only after the surrogacy scandals of 2014 did the Protection of Children Born Through Assisted Reproductive Technologies Act (B.E. 2558) come into effect in 2015, limiting the practice to non-commercial surrogacy between relatives. Exceptions are allowed if a surrogate relative cannot be secured, when appropriate acquaintances can be used instead of a relative. The intended parents must be Thai citizens and a heterosexual couple. International marriage couples with Thai citizen must have been married for more than 3 years prior to engaging in the practice. Approval from the Ministry of Health Committee must be obtained in advance. The commissioning couple is immediately recognized as the legal parents of the child.

As of January 2018, roughly 100 surrogacy cases had been already conducted. The review committee for surrogacy applications consists of 17 members, including obstetricians and gynaecologists, paediatricians, and child welfare specialists (Stasi 2016, 2017). Since the committee was established in 2015, 76 cases have been submitted for review and 72 have been approved. As at January 2018, 140 cases had been approved. Of these, roughly one-third of surrogate mothers were close relatives of the intending parents, one-third were distant relatives, and one-third were non-relatives. According to a government informant, no major problems have been reported (Hibino 2020).

A Thai doctor involved in reproductive medicine evaluated the law in the following way (personal communication, 2018):

Foreigners can no longer request surrogacy in Thailand. However, if you are a Thai citizen, you can. And the conditions are not so strict. If you can't find a surrogate mother from your relatives, friends or any acquaintances are also acceptable. The principle of payment to the surrogate mother will be discussed by the Medical Council of Thailand, but the specific amount has not yet been announced. In my opinion, the surrogate mother will be pregnant for as long as nine months, so some compensation should be granted. With this law, Thai couples can now legally request surrogacy in the country and no longer have to go abroad.

Since the surrogate mother need not be a relative and remuneration has not yet been determined, it is easy to find a surrogate mother. While custody is granted to the intended parents, the child’s right to know their birth mother has not yet been established. Nonetheless, considering that the term “protection of children” is included in the title of the law itself, this sends the message that it is in the child’s best interest to be raised under the custody of the intended parents rather than the surrogate mother.

The view that surrogacy between relatives is altruistic surrogacy is held by Vietnam (Hibino 2015), Cambodia, Nepal, India, and Thailand. In the case of Thailand, the law is flexible and doctors have a great deal of discretion, which raises concerns that the intent of the law may be applied arbitrarily (Hongladarom 2018). As a result of this flexibility, there is concern that the law could lead to de facto commercial surrogacy (Techagaisiyavanit 2016). While commercial surrogacy still takes place underground in Thailand and in neighbouring countries, no protection is offered for surrogates (Attawet et al. 2021).

## The Case of the UK as a Host Country

Although altruistic surrogacy is permitted domestically in the UK, the number of British clients seeking surrogacy overseas has been increasing (Jadva et al. 2021). For those who considered it, the reasons behind not engaging in surrogacy in the UK include a perceived lack of legal framework, limited access to potential surrogates, and preferring to have access to a professional agency to manage the surrogacy process. The official number of overseas surrogacy cases slightly exceeded domestic ones, with children born overseas in 51% of cases (162) and children born in the UK in 43% of cases (136) (Jadva et al. 2021).

A significant turning point in British policy toward cross-border surrogacy came in 2014. Under the guise of “the best interests of the child,” a parental order was issued for a couple who requested commercial surrogacy in India. Since then, parental orders have been granted for British intended parents who request commercial surrogacy overseas.

It has been recognized that the UK’s decades-old surrogacy law, enacted in 1986, is outdated. A report released by a working group requesting a revision of the surrogacy law suggests that the law should be revised as follows: the UK should maintain the principle of altruistic surrogacy; the best interests of the child should be the highest priority; the law should specify how to establish the legal relationship between intended parents and a child born through surrogacy; the parental order should be pre-approved and the client should be registered as a parent at the same time as the birth of the child; and a more detailed definition of compensation for the surrogate mother should be included (Horsey et al. 2015, 2018).

Based on this recommendation from May 2018, the England and Wales Law Commission and the Scottish Legal Commission (2019) began a 3-year review of the amendments to the Surrogacy Agreement Act. The report was published in June of 2019. The consultation deadline was October 2019, with the revised bill to be published in 2022.

The report allows commercial surrogacy abroad while maintaining altruistic surrogacy domestically. Overseas surrogacy might be recommended, which in most cases means commercial surrogacy. Recently, during the COVID-19 pandemic, the UK attempted to make it easier for British intended parents by expediting passport applications and by providing remote parental order hearings. The rights of intended parents may also be expanded in the revised bill, such as being able to gain custody of the child without requiring a parental order. Even though the UK’s revised surrogacy law advocates for an altruistic surrogacy model, in reality, it is a commercial model.

## International Organizations

As concerns surrounding surrogacy tourism have grown, international organizations have begun to conduct their own research. In light of “the best interests of the child”, the surrogacy project conducted by the Hague Conference on Private International Law (called the “Parentage/Surrogacy Project”) is ongoing. Experts in private international law from member countries are attempting to find a universal way to establish legal parent–child relationships when surrogacy is carried out internationally. There are criticisms that such research in itself validates the phenomenon of surrogacy tourism (Hibino et al. 2020).

Lobbying efforts are being carried out within the UN. In a report submitted to the UN in 2019 based on fieldwork conducted in Cambodia (University of Chicago Law School—Global Human Rights Clinic 2019), the following recommendations were made: a permanent ban on surrogacy by the Cambodian government is not desirable, and altruistic surrogacy should be properly regulated. Even in cases of illegal surrogacy, it is recommended that the Cambodian government should hand over the child to the intended foreign parents in line with “the best interests of the child” (surrogacy is criminalized in Cambodia and several brokers and surrogates have been arrested, forcing the Cambodian surrogate mothers in these instances to keep the children to prove that they did not intend to sell them (BBC 2018)). This has been questioned by human rights groups on the grounds that it could damage the surrogate mothers’ lives (Blomberg 2019; Goez 2019)).

In effect, these recommendations actively affirm surrogacy tourism and acknowledge that it will likely result in the commercialization of reproduction and the exploitation of economically vulnerable women on a global scale.

## Conclusion

It is difficult to predict how surrogacy tourism will develop on the international stage going forward. Currently, there are mixed positions and there is no clear pathway for an effective policy. Asian countries, such as India and Thailand, that were once host countries for surrogacy tourism have now refused to allow it, with altruistic surrogacy (between relatives) paving the way for de-facto commercial surrogacy. The exploitation of economically vulnerable groups has not been eliminated despite the official exclusion of foreign nationals. Considering that commercial surrogacy still occurs illegally in Asian countries, there may be pressure from guest countries to re-legalize it.

Despite a lack of consensus on surrogacy, there is no doubt that traditional views of “altruistic” and “commercial” surrogacy are inadequate, that surrogacy is a lucrative business worldwide, and that surrogacy stakeholders are influencing research and public policy decision-making. Stakeholders in commercial surrogacy ought to be carefully excluded from the policymaking process, since their participation is equivalent to a violation of conflict of interest principles. Commercialization has increased in recent years due to networks of cross-border markets related to medical tourism schemes in emerging countries. International organizations ought to deal with these issues by publishing guidelines, and these guidelines should be discussed at a distance from commercial surrogacy stakeholders. We conclude that further commercialization should be prevented carefully by investigating the reality of the surrogacy process.

## References

[CR1] Amar, Aparajita. 2017. Surrogacy Regulation Bill: Parliamentary panel report highlights legislation’s draconian, paternalistic nature. *First Post*, 6 September 2017. https://www.firstpost.com/india/surrogacy-regulation-bill-parliamentary-panel-report-highlights-legislations-draconian-paternalistic-nature-4015097.html. Accessed 13 Sept 2018.

[CR2] Arvidsoon, Anna, Polly Vauquiline, Sara Johnsdotter, and Birgitta Essen. 2017. Surrogate mother-praiseworthy or stigmatized: A qualitative study on perception of surrogacy in Assam, India*. Global Health Action* 10 (1): 1328890. 10.1080/16549716.2017.1328890. 10.1080/16549716.2017.1328890 PMC5496060 28604252

[CR3] Attawet Jutharat (2022). Reconsidering surrogacy legislation in Thailand. Medico-Legal Journal.

[CR4] Attawet Jutharat, Wang Alex, Sullivan Elizabeth (2021). ‘Womb for work’ experiences of Thai women and gestational surrogacy practice in Thailand. Human Fertility.

[CR5] BBC. 2017a. Australian nurse jailed for illegal Cambodian surrogacy. *BBC*, 3 August 2017. https://www.bbc.com/news/world-asia-40810351. Accessed 14 Aug 2022.

[CR6] BBC. 2017b. Thai police arrest man smuggling semen into Laos. *BBC*, 21 April 2017. https://www.bbc.com/news/world-asia-39663671. Accessed 14 Aug 2022.

[CR7] BBC. 2018. Cambodia releases surrogate mothers who agree to keep children. *BBC*, 6 December 2018. https://www.bbc.com/news/world-asia-46466888. Accessed 14 Aug 2022.

[CR8] Bedi, Rahul. 2016. India unveils plan to ban commercial surrogacy. *Telegraph*, 25 August 2016. https://www.telegraph.co.uk/news/2016/08/25/india-unveils-plan-to-ban-commercial-surrogacy/. Accessed 14 Aug 2022.

[CR9] Berkowitz Dana (2007). A sociohistorical analysis of gay men’s procreative consciousness. Journal of GLBT Family Studies.

[CR10] Blomberg, Matt. 2019. Cambodia urged not to criminalize surrogate mothers with new law. *Reuters*, 17 May 2019. https://www.reuters.com/article/us-cambodia-women-lawmaking/cambodia-urged-not-to-criminalize-surrogate-mothers-with-new-law-idUSKCN1SN16S. Accessed 14 Aug 2022.

[CR11] BMJ (2019). Doctors and campaigners oppose India’s proposed surrogacy law. BMJ.

[CR12] Choudhury C. Akila (2016). Transnational commercial surrogacy: Contract, conflicts, and the prospects of international legal regulation. In Oxford Handbook Topics in Law. Oxford: Oxford University Press..

[CR13] Condon, Natalie. 2016. Fragmentation sexuality: the transnational gestational surrogacy market in Thailand and the nationalist “baby gammy” scandal. *Radicle: Reed Anthoropology Review* 1(1). https://radiclejournal.org/index.php/rrar/article/view/13. Accessed 14 Aug 2022.

[CR14] Crozier GKD (2014). Too blunt a tool: A case for subsuming analysis of exploitation in transnational gestational surrogacy under a justice of human rights framework. American Journal of Bioethics.

[CR15] Desai, Kishwar. 2012. India’s surrogate mothers are risking their lives. They urgently need protection. *The Guardian, *5 June 2012. https://www.theguardian.com/commentisfree/2012/jun/05/india-surrogates-impoverished-die. Accessed 14 Aug 2022.

[CR16] DPA. 2017. Cambodia unveils new surrogacy rules. *Qatar Tribune, *6 April 2017. https://www.qatar-tribune.com/article/58116/PHILIPPINES/Cambodia-unveils-new-surrogacy-rules. Accessed 14 Aug 2022.

[CR17] England and Wales Law Commission, and Scottish Law Commission. 2019. Building families through surrogacy: a new law. https://www.lawcom.gov.uk/project/surrogacy/. Accessed 14 Aug 2022.

[CR18] Fronek Patricia, Karen Rotabi S (2020). The impact of the COVID-19 pandemic on intercountry adoption and international commercial surrogacy. International Social Work.

[CR19] Goez, Lea. 2019. UN call to stop criminalization of surrogates in Cambodia. *Progress Educational Trust (PET),* 15 November 2019. https://www.progress.org.uk/un-call-to-stop-criminalisation-of-surrogates-in-cambodia/*.* Accessed 14 Aug 2022.

[CR20] Handley, Erin. 2018. Cambodia: 33 pregnant women found in raid on child surrogacy ring. *The Guardian*, 23 June 2018. https://www.theguardian.com/world/2018/jun/23/cambodia-33-pregnant-women-found-in-raid-on-child-surrogacy-ring. Accessed 14 Aug 2022.

[CR21] Head, Jonathan. 2018. 'Baby factory' mystery: Thailand’s surrogacy sage reaches uneasy end. *BBC*, 26 February 2018. https://www.bbc.com/news/world-asia-43169974. Accessed 14 Aug 2022.

[CR22] Hibino Yuri (2015). Implications of the legalization of non-commercial surrogacy for local kinship and motherhood in Vietnamese society. Reproductive Biomedicine Online.

[CR23] Hibino Yuri (2020). Non-commercial surrogacy in Thailand: Ethical, legal and social implications in local and global contexts. Asian Bioethics Review.

[CR24] Hibino, Yuri, Allan, Sonia and Adams, Damian. 2020. Continuing issues and debate concerning transnational commercial surrogacy during the COVID-10 pandemic and beyond. *Progress Educational Trust (PET)*, 23 November 2020. https://www.progress.org.uk/continuing-issues-and-debate-concerning-transnational-commercial-surrogacy-during-the-covid-19-pandemic-and-beyond/. Accessed 14 Aug 2022.

[CR25] Hongladarom, Soraj. 2018. Surrogacy law in Thailand. ResearchGate. https://www.researchgate.net/publication/322286708. Accessed 14 Aug 2022.

[CR26] Horsey, Kirsty, Natalie Smith, Sarah Norcross, Louisa Ghevaert, and Sarah Jones. 2015. Surrogacy in the UK: Myth busting and reform. Report of the Surrogacy UK Working Group on Surrogacy Law Reform. *Surrogacy UK*, November 2015.

[CR27] Horsey, Kirsty, Natalie Smith, Alan McLeallan, Sarah Norcross, Andrew Powell, and Sarah Jones. 2018 Surrogacy in the UK: Further evidence for reform. Second Report of the Surrogacy UK Working Group on Surrogacy Law Reform. *Surrogacy UK*, December 2018.

[CR28] Indian Council of Medical Research. 2005. National Guidelines for Accreditation, Supervision and Regulation of ART Clinics in India. https://main.icmr.nic.in/sites/default/files/art/ART_Pdf.pdf. Accessed 14 Aug 2022.

[CR29] Jadva Vasanti, Prosser Helen, Natalie Gamble (2021). Cross-border and domestic surrogacy in the UK context: An exploration of practical and legal decision-making. Human Fertility.

[CR30] Johnson Martin (2019). A short history of in vitro fertilization (IVF). International Journal of Developmental Biology.

[CR31] Kalantry, Sital, Rebecca K. Helm, Aparna Chandra, and Mrinal Satish. 2017. Should compensated surrogacy be permitted or prohibited? *Cornell Law School, and National Law University-Delhi*. https://scholarship.law.cornell.edu/facpub/1551/. Accessed 14 Aug 2022.

[CR32] Kirby Jeffrey (2014). Transnational gestational surrogacy: Does it have to be exploitative?. American Journal of Bioethics.

[CR33] Lloyd Parry, Richard. 2014. Billionaire’s son ‘wanted 1000 babies’. *The Times*, 16 August 2014. https://www.thetimes.co.uk/article/billionaires-son-wanted-1000-babies-sczxscgcf2p. Accessed 14 Aug 2014.

[CR34] Mahapatra, Dhananjay. 2008. Baby Manji’s case throws up need for law on surrogacy. *Times of India*, 25 August 2008. https://timesofindia.indiatimes.com/india/baby-manjis-case-throws-up-need-for-law-on-surrogacy/articleshow/3400842.cms. Accessed 14 Aug 2022.

[CR35] Medical Council of Thailand. 2002. Announcement No. 21/2545 on the standards of services involving reproduction technology. https://tmc.or.th/service_law03_7.php. Accessed 14 Aug 2022.

[CR36] Meyers-Belkin, Haxie. 2020. Ukraine’s Covid-19 lockdown leads to baby pileup and surrogacy backlash. *France 24*, 18 June 2020. https://www.france24.com/en/20200618-ukraine-s-covid-19-lockdown-leads-to-baby-pile-up-and-surrogacy-backlash. Accessed 14 Aug 2022.

[CR37] Murdoch, Lindsay. 2014. Australian couple leaves Down syndrome baby with Thai surrogate. *Sydney Morning Herald, *1 August 2014*. *https://www.smh.com.au/national/australian-couple-leaves-down-syndrome-baby-with-thai-surrogate-20140731-zz3xp.html. Accessed 14 Aug 2022.

[CR38] Nadimpally, Sarojini, Sneha Banerjee, and Deepa Venkatachalam. 2016. *Commercial surrogacy: A contested terrain in the realm of rights and justice*. Kuala Lumpur: Asian-Pacific Resource and Research Centre for Women (ARROW). https://arrow.org.my/wp-content/uploads/2018/10/accessible%20pdf-9944/index.pdf. Accessed 14 Aug 2022.

[CR39] Nahavandi Firouzeh (2016). Commodification of body parts in the global south: Transnational inequalities and development challenges.

[CR40] Olaye-Felix, Bianca, Deborah Emma Allen, and Neil H. Metcalfe. 2022. Surrogacy and the law in the UK. *Postgraduate Medical Journal,* 30 June 2022*. *10.1136/postgradmedj-2022-141625. 10.1136/postmj/postgradmedj-2022-141625 37076444

[CR41] Orfali Kris, Chiappori A. Pierre (2014). Transnational gestational surrogacy: Exploitative or empowering?. American Journal of Bioethics.

[CR42] Pande Amrita (2011). Transnational commercial surrogacy in India: Gifts for global sisters?. Reproductive Biomedicine Online.

[CR43] Pande, Amrita. 2013. “At least I am not sleeping with anyone”: Resisting the stigma of commercial surrogacy in India. *Feminist Studies* 36(2): 292–312.

[CR44] Photopoulos, Julianna. 2017. Parliamentary panel suggests liberal reforms to India surrogacy bill. *Progress Educational Trust (PET), *14 August 2017. https://www.progress.org.uk/parliamentary-panel-suggests-liberal-reforms-to-india-surrogacy-bill/. Accessed 14 Aug 2022.

[CR45] Roth, Andrew. 2020. Up to 1,000 babies born to surrogate mothers stranded in Russia. *The Guardian*, 29 July 2020. https://www.theguardian.com/lifeandstyle/2020/jul/29/up-to-1000-babies-born-to-surrogate-mothers-stranded-in-russia. Accessed 14 Aug 2022.

[CR46] Rozée Virginie, Unisa Sayeed, de La Rochebrochard Elise (2020). The social paradoxes of commercial surrogacy in developing countries: India before the new law of 2018. BMC Women's Health.

[CR47] Rudrappa, Sharmila. 2016. Why India’s new surrogacy bill is bad for women. *HuffPost, *26 August 2016. https://www.huffpost.com/entry/why-indias-new-surrogacy-bill-is-bad-for-women_b_57c075f9e4b0b01630de83ad. Accessed 14 Aug 2022.

[CR48] Rudrappa Sharmila (2017). Reproducing dystopia: The politics of transnational surrogacy in India 2020–2015. Critical Sociology.

[CR49] Shetty Priya (2012). India’s unregulated surrogacy industry. Lancet..

[CR50] Stasi, Alessandro. 2016. Maternity surrogacy and reproductive tourism in Thailand: A call for legal enforcement. *Ubon Ratchathani University Law Journal* 8(16): 17–36. https://so06.tci-thaijo.org/index.php/law_ubu/article/view/84719. Accessed 14 Aug 2022.

[CR51] Stasi Alessandro (2017). Protection of children born through assisted reproductive technologies Act, B.E. 2558: The changing profile of surrogacy in Thailand. Clinical Medicine Insights Reproductive Health.

[CR52] Techagaisiyavanit, Wanaporn. 2016. Reproductive justice dilemma under the new Thai law: Children born out of assisted reproductive technology protection act B.E. 2558. *Thammasat Law Journal* 45 (1): 201–214.

[CR53] Timms Olinda (2018). Ending commercial surrogacy in India: significance of the Surrogacy (Regulation) Bill 2016. Indian Journal of Medical Ethics.

[CR54] Timms Olinda (2018). Report of the Parliamentary Standing Committee on the Surrogacy (Regulation) Bill 2016: A commentary. Indian Journal of Medical Ethics.

[CR55] Trompenaars Alfons, Hampden-Turner Charles (1997). Riding the waves of culture; Understanding diversity in global business.

[CR56] Ukrinform. 2020. Children’s ombudsman proposes banning surrogacy in Ukraine. *Ukrinform*, 15 May 2020. https://www.ukrinform.net/rubric-society/3025949-childrens-ombudsman-proposes-banning-surrogacy-in-ukraine.html. Accessed 14 Aug 2022.

[CR57] University of Chicago Law School - Global Human Rights Clinic. 2019. human rights implications of global surrogacy. *Global Human Rights Clinic* 10. https://chicagounbound.uchicago.edu/ihrc/10. Accessed 14 Aug 2022.

[CR58] van Zyl Liezl, Walker Ruth (2013). Beyond altruistic and commercial contract motherhood: The professional model. Bioethics.

[CR59] Vida, Taing. 2018. Surrogacy law progresses. *Khmer Times, *29 November 2018. https://www.khmertimeskh.com/553934/surrogacy-law-progresses/. Accessed 14 Aug 2022.

[CR60] Virutamasen, Pramuan, Kamthiorn Pruksananonda, Khunying Limpaphayom, V. Chokevivat, and Supachai Kunaratanapruk. 2001. The regulation of assisted reproductive technology in Thailand. *Journal of the Medical Association of Thailand* 84 (100): 1490–1494. https://pubmed.ncbi.nlm.nih.gov/11804261/. Accessed 14 Aug 2022. 11804261

[CR61] Whittaker Andrea, Rozée Virginie, Unisa Sayeed (2016). Circumvention, crisis and confusion: Australians crossing borders to Thailand for international surrogacy. *Assisted Reproductive Technologies in the global south and north: issues, challenges and the future*.

[CR62] Whittaker Andrea (2016). From ‘Mung Ming’ to ‘Baby Gammy’: A local history of assisted reproduction in Thailand. Reproductive Biomedicine & Society Online.

